# A case of massive primary tumor growth in the immediate postoperative period

**DOI:** 10.1002/ccr3.2679

**Published:** 2020-01-28

**Authors:** Konstantinos A. Boulas, Aikaterini Paraskeva, Alexandros Triantafyllidis, Anestis Hatzigeorgiadis

**Affiliations:** ^1^ Department of General Surgery General Hospital of Drama Drama Greece

**Keywords:** antiangiogenic therapy, surgery, thrombocytopenia, tumor growth

## Abstract

Major surgical trauma along with discontinuation of antiangiogenic treatment can exacerbate primary tumor growth even in the immediate postoperative period.

## Graphical abstract

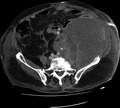

An otherwise‐healthy 71‐year‐old male patient with a recurrent stage IV (G2, cT4cN0cM1) left retroperitoneal malignant fibrous histiocytoma under pazopanib was admitted with signs of peritonitis. CT showed a 16 × 10 × 12cm left retroperitoneal mass with descending colon infiltration and perforation (Figure 1). Left hemicolectomy with end transverse colostomy and without tumor resection performed. Postoperatively, pazopanib discontinued, and tinzaparin and imipenem administered. Regarding surgical complications, postoperative period was uneventful. On the 19th postoperative day, isolated severe thrombocytopenia (PLT <10 x 10^9^/L) and mild coagulopathy developed (fibrinogen 198 mg/dL, D‐dimmer 4.25 μg/mL). CT revealed progressive disease with massive primary tumor growth measuring 20 × 14 × 15cm with 2.5‐fold volume increase (Figure 2).

**Figure 1:**
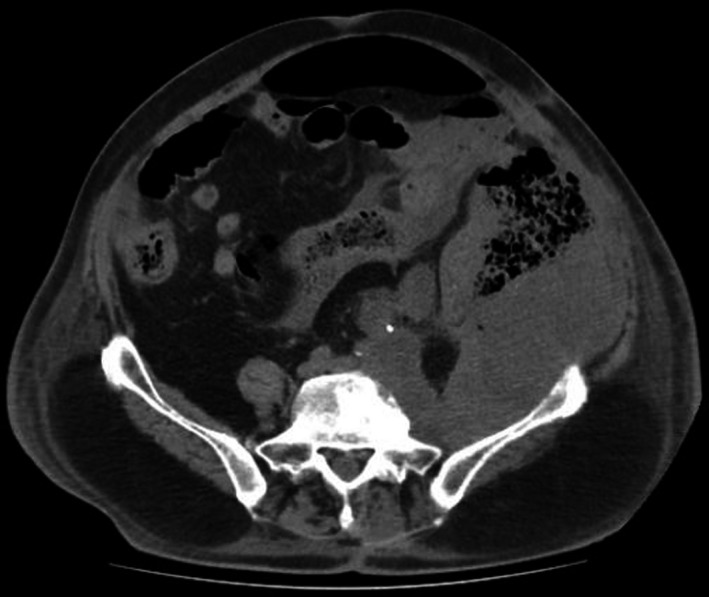
CT revealed the presence of a 16 × 10 × 12 cm solid left retroperitoneal mass with infiltration and perforation of the descending colon

**Figure 2:**
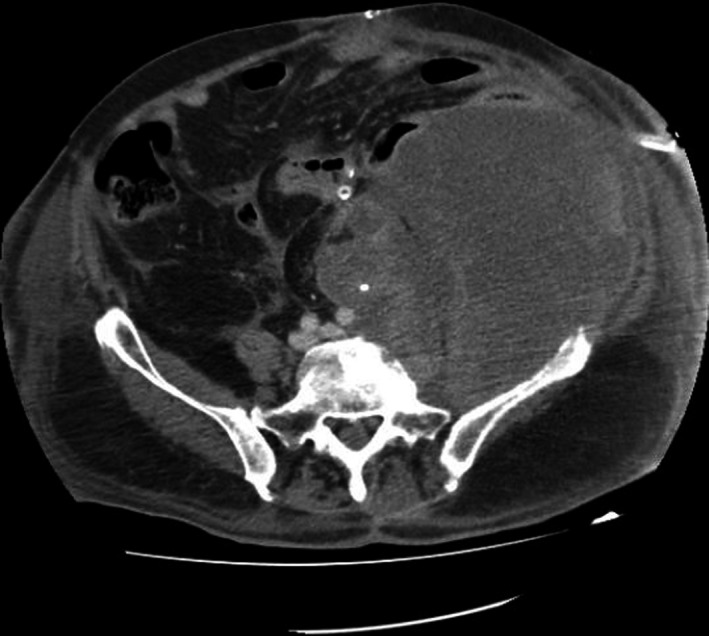
Postoperative CT showed rapid and massive primary tumor growth measuring approximately 20 × 14 × 15 cm in size with a 2.5‐fold increase in volume

## 1 QUIZ QUESTION: WHAT TRIGGERED THE MASSIVE TUMOR GROWTH?

Surgical trauma can influence pathophysiological processes, such as wound healing response, local and systemic immunosuppression, that promote postoperative metastatic spread and recurrence.^1^ Discontinuation of antiangiogenic treatment can cause increase in tumor growth, which is higher in patients with discontinuation due to disease progression and lower in patients with sustained response.^2^ In our patient's case, major surgical trauma and VEGFR‐inhibitor discontinuation resulted in massive primary tumor growth. As other causes of isolated thrombocytopenia (sepsis, drugs, ITP, HIT, and DIC) were excluded, concomitant presentation of massive increase in primary tumor volume and thrombocytopenia was suggestive of platelet trapping and fibrinogen consumption within the abnormal tumor vascularity.

## 2 STATEMENT OF HUMAN AND ANIMAL RIGHTS

The present article does not contain any studies with human or animal subjects performed by any of the authors.

## CONFLICT OF INTEREST

The authors declare that they have no conflict of interests.

## AUTHOR CONTRIBUTION

All authors equally accessed the data and contributed to the preparation of the manuscript. BKA and HA: were equally responsible for making and performing treatment decisions. HA: reviewed the manuscript for critical intellectual content and had the final approval.

## INFORMED CONSENT

Informed consent was obtained from the patient.
